# LSPpred Suite: Tools for Leaderless Secretory Protein Prediction in Plants

**DOI:** 10.3390/plants12071428

**Published:** 2023-03-23

**Authors:** Andrew Lonsdale, Laura Ceballos-Laita, Daisuke Takahashi, Matsuo Uemura, Javier Abadía, Melissa J. Davis, Antony Bacic, Monika S. Doblin

**Affiliations:** 1 ARC Centre of Excellence in Plant Cell Walls, School of BioSciences, The University of Melbourne, Melbourne, VIC 3010, Australia; 2 Plant Stress Physiology Group, Plant Nutrition Department, Aula Dei Experimental Station, CSIC, P.O. Box 13034, 50080 Zaragoza, Spain; 3 United Graduate School of Agricultural Sciences, Iwate University, Morioka 020-8550, Japan; 4 Faculty of Agriculture, Iwate University, Morioka 020-8550, Japan; 5 Bioinformatics, Walter and Eliza Hall Institute for Medical Research, Melbourne, VIC 3052, Australia

**Keywords:** unconventional protein secretion, leaderless secretory proteins, subcellular localisation prediction

## Abstract

Plant proteins that are secreted without a classical signal peptide leader sequence are termed leaderless secretory proteins (LSPs) and are implicated in both plant development and (a)biotic stress responses. In plant proteomics experimental workflows, identification of LSPs is hindered by the possibility of contamination from other subcellar compartments upon purification of the secretome. Applying machine learning algorithms to predict LSPs in plants is also challenging due to the rarity of experimentally validated examples for training purposes. This work attempts to address this issue by establishing criteria for identifying potential plant LSPs based on experimental observations and training random forest classifiers on the putative datasets. The resultant plant protein database LSPDB and bioinformatic prediction tools LSPpred and SPLpred are available at lsppred.lspdb.org. The LSPpred and SPLpred modules are internally validated on the training dataset, with false positives controlled at 5%, and are also able to classify the limited number of established plant LSPs (SPLpred (3/4, LSPpred 4/4). Until such time as a larger set of bona fide (independently experimentally validated) LSPs is established using imaging technologies (light/fluorescence/electron microscopy) to confirm sub-cellular location, these tools represent a bridging method for predicting and identifying plant putative LSPs for subsequent experimental validation.

## 1. Introduction

Secretory proteins typically contain an N-terminal signal peptide (SP), a short region of amino acids with a defined hydrophobic motif [1]. This ‘leader’ sequence is recognised and facilitates the co-translation of the protein into the endoplasmic reticulum (ER) by bound ribosomes (rough ER). This marks the start of the classical/conventional secretory protein (CSP) pathway through the ER, Golgi apparatus (GA), to the cell surface (either the plasma membrane (PM) and/or the extracellular space (apoplast/cell wall)), with specific motifs (generally within the protein sequence) acting as “post codes” for retention in a particular sub-cellular compartment; in the absence of a “post code”, secretion of the (glyco)protein defaults to the PM/cell surface. However, an understanding of alternative methods for protein secretion that deviate from the conventional secretory pathway is emerging.

Any deviation from the CSP is defined as unconventional, a term that encompasses several possible mechanisms for secretory trafficking. Primarily, it is deviation from the conventional protein secretion pathway that has given rise to the names of these alternate routes—non-classical, non-conventional, unconventional, and leaderless. Leaderless, in this case, refers to proteins without an SP, called leaderless secretory proteins (LSPs), and the broader process they undergo is unconventional protein secretion (UPS). This broad definition allows for other forms of UPS that have been shown to still involve either an SP or TMD sequence, for example, when proteins enter the ER via the classical mechanism but bypass the Golgi or *trans* Golgi network (TGN) in their journey to the apoplast [2].

Eukaryotic UPS is best understood in mammals and yeast [3,4], and although examples of UPS are known from *Drosophila melanogaster* and *Leishmania major* [5] they are, in general, poorly understood in plants. The proposed UPS pathways derived from these studies have been reviewed [6,7,8], although, in most cases, the molecular mechanisms are still poorly understood [9]. Currently, the defined categories are: Type I, pore-mediated secretion across the PM; Type II, ABC transporter-based secretion; Type III, autophagy or organelle related; and Type IV, Golgi bypass (not necessarily LSPs). Recent work on extracellular vesicles (EVs) has labelled this avenue of protein secretion as a UPS pathway for LSPs [9], and though similar to Type III, suggested it should be defined as a distinct pathway. In plants, the UPS contents of EVs have been suggested to be due to these autophagy- or organelle-related pathways but define the creation of EVs as a distinct step [7]. Another UPS review, focussed on mechanisms of human diseases [10], reduced the Type I-IV classification system to three pathways: leaderless non-vesicular UPS (combining Type I and Type II from [6], leaderless vesicular UPS, and the Golgi bypass route (Type IV). These broad categories match pathways proposed in plant-specific reviews [7,11], except for unconventional vacuole delivery, which could be considered another form of UPS.

Exemplar proteins exist, mostly in mammals and yeast, for each of the four pathways. While there are constitutively expressed LSPs (e.g., human fibroblast growth factor 2 (FGF2) [5], stress conditions (disease and/or environmental) are thought to largely contribute to UPS. For example, in mammalian systems, stress such as inflammation leads to leaderless secretion of interleukin 1(IL-1 β), transglutaminase 2 (TG2), and thioredoxin (THX) [6] through pores (Type I). In the human immuno-deficiency virus (HIV), the transactivator of transcription (TAT) protein is also proposed to constitutively make pores for UPS [5] in order to avoid modifications that would occur in the GA, such as glycosylation.

UPS is a well-established phenomenon in bacteria, although often different classification systems are used, and the various non-SP-mediated secretion pathways are not always referred to as unconventional [12]. Early work defined bacterial UPS as secretion that did not follow either conventional Sec or Tat (twin-arginine translocation) secretion [13]. However, there are now at least 12 categories of secretion defined in bacteria [14]. Since these do not involve membrane-bound organelles, they are most similar to Type I UPS mechanisms in eukaryotes. A recent study re-evaluated non-classical secretion systems and led to the development of a new prediction tool for UPS in Gram-positive bacteria, PeNGaRoo [15].

Despite the focus on mammalian and yeast UPS, there is growing evidence and interest in plant LSPs and UPS [16]. UPS in plants is thought to be maintained as both an alternative pathway for constitutive secretion as well as for responding to stress and microbial symbiosis [2,3,7,16,17,18]. Distinguishing between CSP and contamination is difficult in plant apoplast/secretome studies. Up to 50% of proteins found in the plant cell wall/apoplast secretomes through sub-cellular fractionation studies lack an SP [19,20]. All these proteins are unlikely to be LSPs as the levels of intracellular contamination are generally quite high using these experimental approaches. Bioinformatic prediction tools can potentially assist by identifying putative LSPs.

Prediction of putative LSPs is most commonly performed using SecretomeP [21] but as it has not been trained on plant data, we and others have shown that its predictive capacity of plant protein secretion is extremely poor [12,22]. The hypothesis underlying SecretomeP is indirect; it uses purported common features between CSPs and LSPs by training on CSPs with their SP removed. A more direct computational approach would be to train the program on the features of LSPs directly, but this is problematic for plants as there are very few experimentally verified LSPs. Mannitol dehydrogenase (MDH) in celery (*Apium graveolens*) is amongst the few plant proteins generally accepted to undergo UPS [16,23], as it was found to be secreted after the application of salicylic acid, which simulated a response to pathogens, and its secretion is not inhibited by brefeldin A, a chemical that disrupts conventional, Golgi-dependent trafficking.

The multivesicular body (MVB) pathway (Type III) is implicated in the secretion of the best characterized plant LSP, Helja [24,25] from sunflower (*Helianthus annuus*). Another Type III or vesicular UPS-mediated protein is S-adenosylmethionine synthetase (SAMS2, AT4G01850), an LSP shown to co-locate with the exocyst-positive organelle (EXPO) using an SAMS2-GFP construct in *Arabidopsis thaliana* [26]. The extracellular functions of the EXPO-mediated secretory proteins are not yet clear. Adding to these three plant candidates is the bacterial hygromycin B phosphotransferase (HYG^R^), which inactivates hygromycin B. Despite it being an antibiotic resistance protein from *Escherichia coli,* it has been included in plant UPS reviews [3,16]. UPS has been demonstrated for heterologously expressed HYG^R^ in transgenic *Arabidopsis* and *Nicotiana benthamiana* plants, whereby the extracellular localisation of an HYG-GFP fusion construct (lacking an SP) was used to infer leaderless secretion [27,28]. The inclusion of HYG^R^ from *E. coli* in the plant LSP literature is questionable, and similarly, whether to consider it as an example of plant leaderless secretion for this work needs consideration. Creating a new prediction tool to replace SecretomeP would typically require many more ‘true positive’ proteins to train a classifier than these few examples, including ones with convincing experimental evidence.

**Figure 1:**
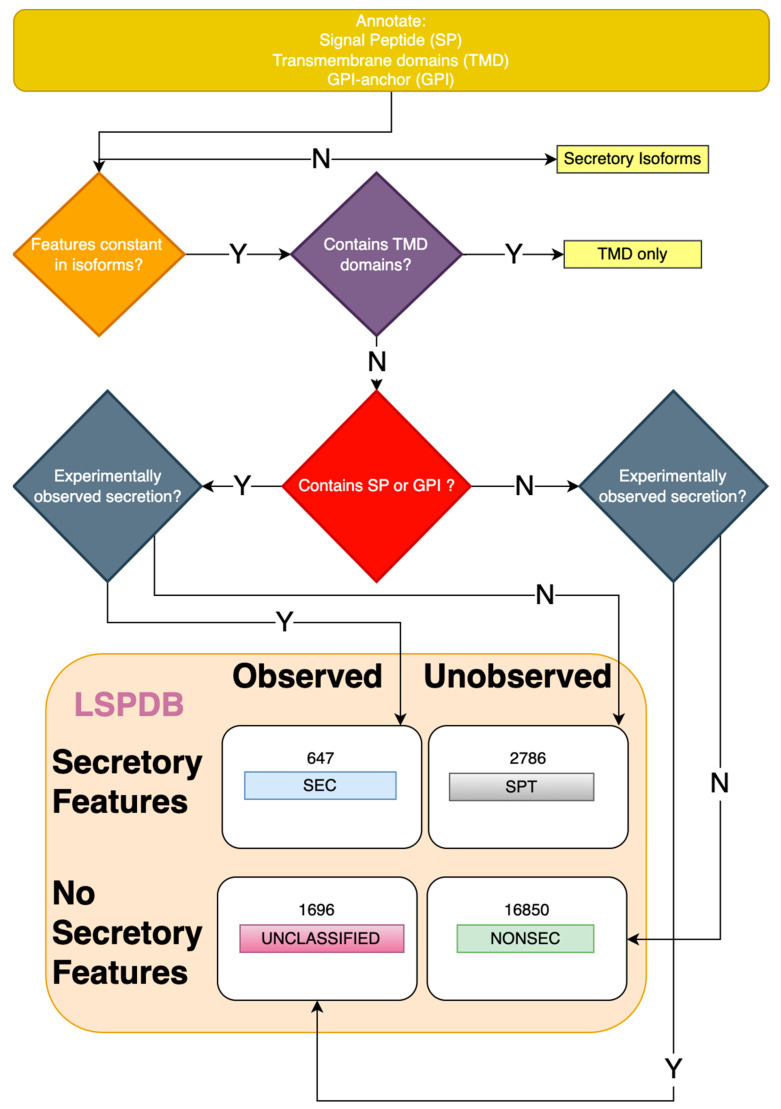
LSP database (LSPDB) workflow. Schematic overview of the LSPD workflow, using an entire plant proteome (*A. thaliana*) as input and annotating each protein isoform with known secretory features, including a signal peptide (SP), transmembrane domains (TMDs) and GPI anchor. Protein isoforms derived from the same gene are compared for differing secretory features to identify genes that have both secretory and non-secretory protein products, as these are not guaranteed to be able to be confidently assigned to either category. These are labelled as ‘secretory isoforms’ and not categorised further. Genes with only TMDs are also excluded from classification and are labelled ‘TM only’. The remainder are then classified based on whether they have or lack any secretory features and if they are observed or unobserved in plant cell wall/secretome papers (see Supplementary Table S1) included in the LSPDB. The combinations of classification lead to four final category labels (totals shown for *Arabidopsis*): observed classically secreted proteins or SEC (647), classically secreted proteins not observed or SPT/SP_THEORY (2786), proteins observed that lack secretory features or UNCLASSIFIED (1696), and the majority of proteins that are unobserved and lack secretory proteins NONSEC (16850).

To break this impasse, the work presented here seeks to build a classifier by curating sets of putative plant LSPs, based on the features of proteins observed in proteomic studies of the plant cell surface (PM/cell wall). Probable plant LSPs are identified using defined criteria based on a combination of secretory features and observations in selected proteome experiments (Figure 1). Proteins from *Arabidopsis* were used as the source for these observations due to the large number of proteomic resources and rich genomic/proteomic annotation of this model plant. The entire *Arabidopsis* proteome was then categorised into an LSP database (LSPDB) based on each protein isoform’s secretory features and whether the proteins have been experimentally observed in secretomes. Features of these observations were used to create a rule-based approach to identify likely LSPs in LSPDB. The LSPDB data were then used to build a random forest classifier, called LSPpred, of likely LSPs against expected non-secreted proteins. Additionally, the observed CSPs in LSPDB were used to replicate the SecretomeP common feature hypothesis in a plant-specific setting, using the same random forest model, to create a sub-module of LSPpred called SPLpred. Internal validation of the tools estimated a false-positive rate of less than 0.05, and application to the limited set of generally accepted plant LSPs yielded promising results. The results of either LSPpred or SPLpred modules can be used to filter and prioritise potential LSPs. Ultimately, this tool should be superseded by computational approaches based on the features of bona fide LSPs that have been experimentally validated using imaging (light/fluorescence/electron microscopy) approaches to verify their sub-cellular location with protein-specific antibodies and/or heterologously expressed tagged proteins.

## 2. Methods

### 2.1. Generation of a Leaderless Secretory Protein Database (LSPDB)

**Figure 2:**
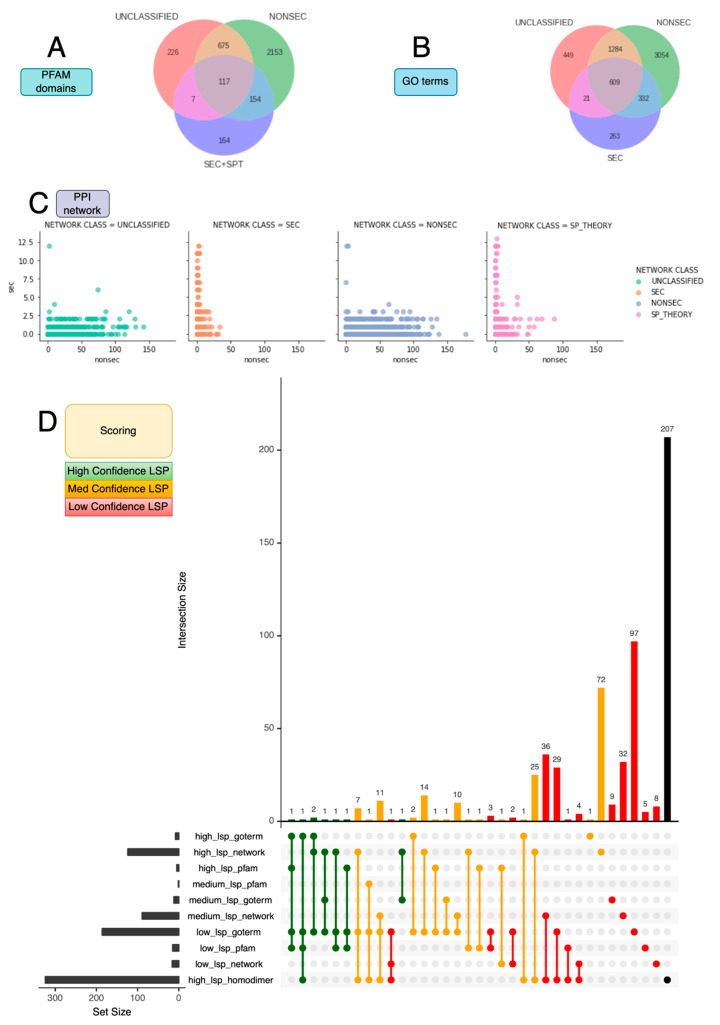
LSPDB Scoring. Overview of the individual evidence categories. (**A**) Venn diagram of common PFAM domains from the combined SEC/SPT, NONSEC, and UNCLASSIFIED LSPDB classes. (**B**) Venn diagram of common GO terms from the combined SEC/SPT, NONSEC, and UNCLASSIFIED LSPDB classes, and (**C**) profile of SEC and NONSEC interactions of each LSPDB class according the LSPDB PPI network. (**D**) UpSet plot of the overlapping proteins that meet the individual confidence criteria as outlined in Table 1 to form the high-confidence set (green—8 proteins), medium (orange—149), and low (red—227). A further 207 (black) are noted as homodimers without any other evidence.

Curated training data for LSP classifiers was drawn from a custom Leaderless Secretory Protein Database (Figure 1). Briefly, relevant secretory features were predicted for the entire *Arabidopsis* proteome and protein isoforms labelled if they contained (or lacked) classical secretory features, such as a signal peptide (SP), for entry into the conventional secretory pathway [29], transmembrane domains (TMDs), and GPI anchors (GPI) for embedding or attachment, respectively, to the PM. These predictions were used to capture all relevant features typical of secretion in a high-throughput manner, irrespective of existing annotations. These labels were then combined with observations from relevant proteomic literature and four categories specified. This included observed classically secreted proteins (SECs), classically secreted proteins not observed (SPT/SP_THEORY), proteins observed that lack secretory features (UNCLASSIFIED), and the majority of proteins that are unobserved and lack secretory features (NONSEC). Proteins with the UNCLASSIFIED label are candidates for LSPs, and the similarity of these proteins to the other categories was used to create a set of tiers and scoring criteria for training data for a classifier (Figure 2). This stratified the UNCLASSIFIED proteins into high-, medium-, and low-confidence LSPs under each of three criteria: network interactions, GO terms, and PFAM domains. The combination of these individual confidence scores was then used to assign an overall high, medium, or low LSP score for the UNCLASSIFIED data (Table 1). See Supplementary Methods for further details.

### 2.2. Input Data for Classifiers

**Figure 3:**
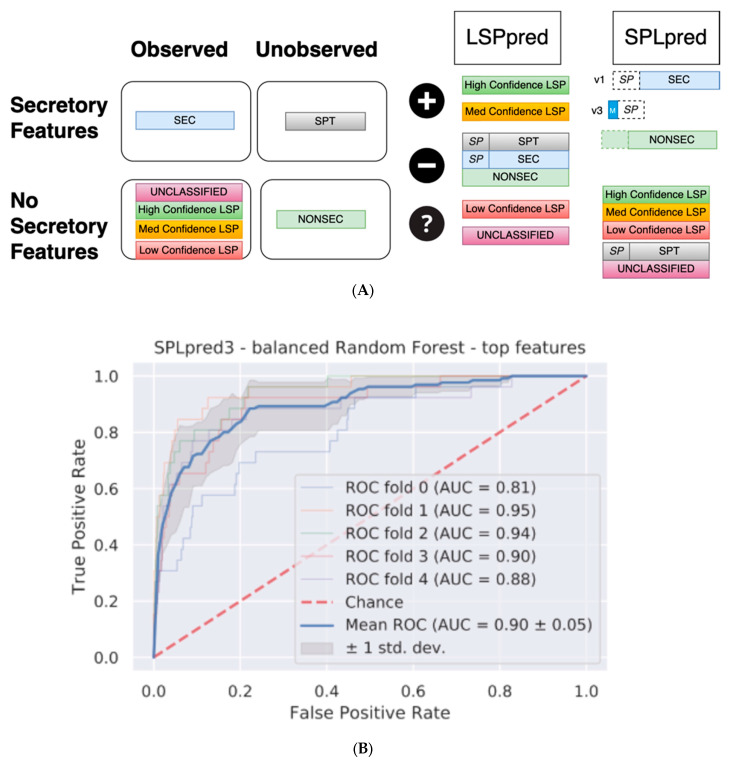

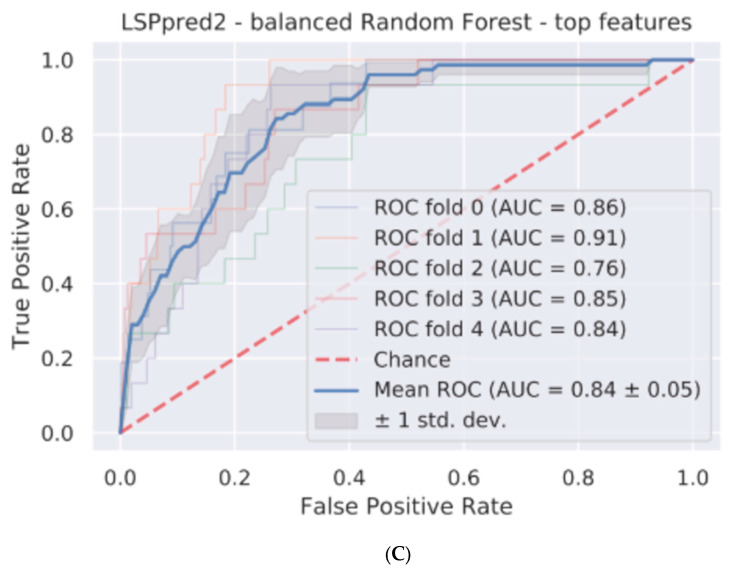
LSPpred and SPLpred. (**A**) Training data used for LSPpred and SPLpred, with subsets of the LSPDB classifications indicated as members of observed or unobserved, and secretory features or no secretory features. The application for SPLpred and LSPpred is also shown, with each subset labelled according to its use as positive data (+ symbol), negative data (− symbol) or evaluation data (? symbol). Modifications to the SP region are shown with dotted lines. (**B**,**C**) The results of LSPpred and SPLpred on held-out test data, with five-fold cross validated ROC scores and mean ROC annotated for the final random forest models, are shown in (**B**) SPLpred3 (AUC 0.90) and (**C**) LSPpred2 (0.84), respectively.

The LSPDB classifications were used to build random forest classifier modules for LSP prediction. Each module uses different LSPDB data and a different approach to tackle the problem of LSP prediction in plants (Figure 3). These approaches led to different combinations of the LSPDB data being selected as positive and/or negative data (Figure 3A). The choice of input data can also guide the mode of operation of the predictors, so multiple versions of each module were generated. The LSPpred module uses putative LSPs in the UNCLASSIFIED high and medium category as positive training data, against both the classically (SEC) and non-secreted (NONSEC) data to use features unique to the putative LSPs as the basis for prediction. Low-confidence and the remaining UNCLASSIFIED proteins are reserved as evaluation data and were not included in the training. Two versions of the classifier were compared (version 1, with only NONSEC data as the negative, and version 2, with SEC and SPT proteins as additional negative training data (LSPpred1 and LSPpred2, respectively; Supplementary Figure S3)). The second module, SPLpred, mimics the SecretomeP hypothesis with a signal peptide removed from CSPs, and these positive modified data are trained against NONSEC proteins as negative data (Figure 3). It should be emphasised that these data are from *Arabidopsis*, as opposed to the human data in the eukaryotic version of SecretomeP. Four versions of this classifier were constructed (Supplementary Figure S1): version 1 with SP removed from the positive data (SPLpred1); version 2 removed either the SP or amino acids of average SP length from the positive and negative data as appropriate (SPLpred2); version 3 removed SP from positive data with the translation-initiating methionine residue restored (SPLpred3); and version 4 used duplicated SP removed and intact versions of these data (SPLpred4). The modifications were performed using text operation on protein sequences, removing either the annotated SP regions or an equivalent length equal to the average of observed SP in cases where negative data were modified. In cases where a leading methionine residue was restored, this was added back to the protein after the removal of the SP. The schematic of these modifications is shown in Supplementary Figure S1, with dotted lines representing the modification areas of the sequences.

For each prediction module, the relevant proteins from the LSPDB gene classes were extracted in FASTA format. Given the possible “one to many” relationships between genes and proteins, initially, all proteins from a classified gene were selected. Several ribosomal LSPs in the medium confidence lists were excluded, as, on inspection, a single protein–protein interaction (PPI) led to a ribosomal complex being included. To avoid a bias and over-reliance on similar sequences (either from the same gene or gene family), CD-hit (version 4.6.8) [30] was used to select protein sequences with up to 40% maximum identity in each class. This is similar to the SecretomeP division into non-similar sets with approx. 26% identity between them for cross-validation. A threshold of 40% was selected to balance basing the predictions on independent data with the limited original data size. The sequence similarity measure ensures the datasets are dissimilar and prevents accuracy from one motif overly influencing the data. After similarity selection, the inputs of each class were reduced to 322 SEC, 1178 UNCLASSIFIED, 1439 SPT, and 10,523 NONSEC proteins. These selected proteins were, henceforth, used as representative for their class. Creating numerical representations of features of the training sequences was performed using the ProFET [31] suite of tools. An initial set of 203 categories, with a total of 1170 scaled features, was derived for the inputs. These categories include properties of sequence length, weight, isoelectric point, grand average of hydropathy (GRAVY, calculated as sum of amino acid hydropathy values divided by length), amino acid compositions, composition based on compressed amino acid alphabets that replace related amino acids with a single value, possible sites of PTMs, autocorrelation, as well as scaled and transformed features.

### 2.3. Training Random Forest Models on Unbalanced Data

Though the positive and negative data were defined separately for the LSPpred and SPLpred approaches, they share the common goal to classify LSPs. Both also use the same large number of initial protein feature representations (ProFET), without prior knowledge of which features are biologically relevant. Both also include the NONSEC data as a negative dataset, which includes the majority of proteins in the LSPDB and ensures that the data are unbalanced; there are many more negative examples than positive. There are many machine learning models for classification that have been applied to bioinformatics, including support vector machines, neural networks, and random forests [32]. Decision trees consist of cascading nodes of tests that lead to classifications, with each node based on the input features. A useful property of random forests for LSPs is that the features can be measured by their Gini importance (or simply importance) to the trees and could be used to infer biological meaning and suggestions for future experimental work. Another key consideration is the source of input data. For LSPpred, the LSP data are selected from the LSPDB, which is based on experimental observation and protein features, rather than from a large set of experimentally validated LSPs. Therefore, a general-purpose machine learning model, such as random forests, is appropriate to explore predicting in this space over more complicated methods.

The random forests were created using the Python scikit learn implementation (v0.19 [33]). However, random forests generalise poorly when trained on unbalanced data. To overcome this limitation, the imbalanced learning extension Balanced Random Forest Classifier (BRF) [34] was used. The input data for each version of SPLpred/LSPpred were split into training and test sets (75% train, 25% test), with stratification to ensure that the relative proportion of positives and negatives was maintained between the training and test data. The BRF set was then trained using 5-fold cross-validation to estimate the accuracy.

### 2.4. Metrics for Preferred Classifier Model Selection

Aiming for the convention of a false-positive rate (FPR) of 0.05, from the cross-validated receiver operator curve (ROC), we estimate a prediction threshold by taking the mean of the 5 thresholds that do not exceed 0.05 FPR across the folds. We can also estimate the true-positive rate (TPR) at this threshold by taking the mean TPR at the same thresholds. Cross-validation also identified the most important features, and for each predictor, these were ranked and the upper quartile selected. The entire dataset was then trained using the BRF model on the reduced set of features. The cross-validated threshold is then used to calculate the accuracy of the complete model on the excluded test data by making a positive prediction for any value exceeding it. A lower confidence classification, untethered to a desired FPR, can also be taken by using a cut-off of 0.5 as the threshold.

Given the class imbalance with fewer positive data points in each version of the predictors, the balanced accuracy, defined as the average of the accuracy of positive and negative data, was used on the test data. Using this metric to compare the versions ensures that performance on scarce positive data is equally weighted with being able to correctly identify true negatives. To estimate bias in the modified predictors of SPLpred, each trained model was then applied to two versions of the SPT dataset, with and without the SP removed. The kernel density estimation (KDE) plot of both prediction distributions was used, and the mean difference in prediction scores for each protein, with and without SP, was calculated. A similar test was performed using any modified or unmodified versions of the training data; for example, if SP-removed data were used as training input, then scores on full-length versions of the sequences were used as a comparison. Since these data have been seen by the model, any deviation would be an indication of bias due to input modifications. These bias and accuracy metrics were used to compare between the alternative designs of the prediction tools and select a preferred candidate for both LSPpred and SPLpred.

## 3. Results

### 3.1. Performance and Relevant Features of the Preferred LSPpred Model

The performance metrics for each of the models are shown in Table 2. Superficially, the area under the ROC (AUROC) and the balanced accuracy results on test data can be used to select the best LSPpred and SPLpred model, respectively. SPLpred, however, is designed for SP protein-specific features using modified SP data, and the conclusions from our previous work [22] support using bias estimates when comparing the predictors. Usefully, neither version of LSPpred used modified proteins for input data, and the bias estimates are expected to be minor. Calculating the difference in prediction scores of modified and unmodified datasets results in a mean difference of 0.03 to 0.05 for all sets (Supplementary Figure S3), which will affect the prediction outcome only in edge cases where the prediction score is within ±0.05 of the thresholds and could cross the boundary from positive to negative prediction or vice versa. Given the lack of modification in training data, these results serve as a baseline guide for differences in predictors; that is, without any modification, there are still some observed differences that could arise from some protein features occurring in the N-terminal region of the protein, and these differences can be compared to predictors that do specifically include modified data. Notably, for LSPpred2, each of the SP, SPT, and NONSEC proteins were included in the training data. The two LSPpred versions have similar AUROC and are highly correlated (Pearson’s *r* = 0.878); however, LSPpred2 in training has observed a variety of data (i.e., inclusive of SP and SPT proteins) and is, therefore, trained to differentiate between LSPs and both NONSEC- and SP-containing proteins. Given this added complexity without loss of accuracy, it is the favoured version, since the unknown inbuilt features of the model were trained in the context of proteins, with SP as negative data. The aim is that the tool does not recognise common aspects of CSPs as a signal for LSPs, which is a more appropriate assumption for the SPLpred models. Therefore, LSPpred2 was selected as the preferred version of the model.

Using the AUROC and test results alone, a superficial conclusion would be that SPLpred1 is the most accurate. Reminiscent of our earlier study [22], the accuracy of SPT proteins has a noticeable shift to lower scores for some proteins (Supplementary Figure S2). Furthermore, the accuracy in the negative training data (which was unmodified) shifts to higher scores when modified. Since this modification will resemble the cleaved SP training data, the suspiciously high accuracy of SPLpred1 seems to be driven by the modifications and the absence of the SP (and, therefore, the translation-initiating methionine residue) is recognised as a feature of secretory proteins. The mean score difference for all sets is also between 3- and 10-times greater than the LSPpred baselines, and so the modifications in SPLpred1 systematically bias the results. SPLpred2 was designed to overcome bias by modifying both the positive and negative data, removing the SP from the positive data and a sequence of average SP length from the start of negative data sequences. Importantly, the bias shift reduced with the mean difference between scores 0.1 (SP), 0.05 (SPT), and 0.06 (NONSEC) (Supplementary Figure S1), which is lower compared to SPLpred1. Tempering the modifications by changing both positive and negative data does reduce the bias, at the cost of modifying more data. LSPpred3, which restores the translation-initiating methionine residue to the positive data, only has an AUROC of 0.90 ± 0.05 (Table 1, Figure 3), which is comparable with LSPpred2. The test data results have a balanced accuracy of 0.82 (35/43) and 0.76 (24/43) when using the threshold (Table 1, Figure 3), which are also broadly similar. The bias distribution is also similar, and, on average, the difference in score is lower than for SPLpred2, with 0.07 (SP), 0.04 (SPT), and 0.04 (NONSEC) (Figure 3). SPLpred4 utilised another approach, doubling the training data with modified and unmodified versions of positive and negative data. Though the accuracy is reasonably high, the bias estimates are the second highest after version 1, with average differences between modified and unmodified scores of 0.15 (SP), 0.06 (SPT), and 0.1 (NONSEC) (Supplementary Figure S1). Although there is similar accuracy between SPLpred2 and SPLpred3, the bias differences are the lowest of the set and comparable with those of LSPpred2, a tool not reliant on modifications. SPLpred3 was, therefore, selected as the preferred version of the model.

These two preferred models, SPLpred2 and SPLpred3, will now be referred to as LSPpred* and SPLpred*, respectively. For each model, we set two modes of operation: high confidence and low confidence. In the high-confidence mode, the threshold (LSPpred*: 0.68, SPLpred*: 0.70) estimated to control the FPR to around 0.05 is used for a positive prediction. Any prediction score either equal to or exceeding the threshold is taken as a positive prediction for secretion. In low-confidence mode, a majority prediction of 0.5 or more indicates that the model preferentially identifies them as LSPs, yet the FPR is expected to be higher in such cases.

### 3.2. Testing on Plant LSPs

The lack of well-characterised plant LSPs has motivated approaches to use either proxy data (such as CSPs) or putative data (such as the LSPDB confidence lists). The cross-validated accuracy scores control for FPR at 5% and test estimates provide some measure of the performance of LSPpred and SPLpred but are not a substitute for independent data. With the caveats that there are too few examples to be statistically confident, we can apply these tools to the best LSP candidates in plants. Importantly, these limited data are independent of the training data in these tools. Two of the candidates (MDH (celery); Helja (sunflower)) are not from *Arabidopsis*, and HYG^R^ is from *E. coli.* SAMS2 in *Arabidopsis* is in the low-confidence list and, therefore, is unseen by both LSPpred and SPLpred and somewhat independent.

We can apply LSPpred* and SPLpred* to these four protein sequences, along with other relevant tools for comparison (Table 3). Amongst the LSP prediction programs published, SRTpred [35] is one of the few still available online and functioning, along with SecretomeP. The tools compared are predictors for LSPs (LSPpred*, SPLpred*, SRTpred and SecretomeP), general sub-cellular localisation (DeepLoc [36]), and apoplastic proteins from either a plant or pathogen source (ApoplastP [37] and EffectorP [38]). Recent UPS-specific programs, such as OutCyte [39] and ExoPred [40], were also included. LSPpred* predicts all four proteins to be LSPs (Table 3). SPLpred*, in high-confidence mode, predicts 50%, noting that with the addition of the low-confidence mode, this increases to 75%. Notably, SecretomeP does not perform well on these proteins. Interestingly, Helja is predicted to be an LSP by both ApoplastP and SRTpred, noting that for ApoplastP, in particular, a plant effector model was used to train the program, independent of SP. It is, therefore, more likely to identify pathogen-related LSPs. DeepLoc is a general tool and gives scores for multiple locations. None of the four proteins were predicted to be apoplastic, and the individual extracellular prediction scores were all low.

Predicting all four plant LSPs by LSPpred* is a promising outcome, yet it is based on too few samples for rigorous assessment of the value of this tool. Since the underlying model is based on putative LSPs, CSPs are not an appropriate proxy for testing its accuracy. Ultimately, increasing the numbers of verified plant LSPs from species other than *Arabidopsis* will be required to evaluate the accuracy and false-positive rate. However, the ability to predict these independent test data combined with the internal cross-validated TPR of 35% with an FPR at 5% indicates that LSPpred* can identify some plant LSPs with acceptable estimates of error. Furthermore, since HYG^R^ is not a plant LSP, this protein highlights that the use of LSPs from other systems could shape future experimental designs, testing which LSPs maintain unconventional secretion between organisms, and possibly revealing common secretion mechanisms. It is also another independent test, with evidence for protein secretion in *Arabidopsis* yet independent of the *Arabidopsis* proteome and, therefore, separate from the data used to create LSPpred*.

### 3.3. An Independent Test of LSPpred and SPLpred Using Experimental Data

Due to the small number of verified plant LSPs, we also sought an independent experimental dataset to demonstrate the use of SPLpred and LSPpred modules. As described in previous work, the proteome of the xylem sap of tomatoes exposed to excess manganese (Mn) was used to investigate the effects of Mn toxicity [41]. Proteins both with and without an N-terminal SP were observed in the xylem sap and assigning them as being secreted either conventionally or unconventionally, or because of contamination from adjacent tissue, was essential. The abundance of 668 candidate proteins across six replicate studies was used to identify 322 proteins with statistically significant differences (≥50% decrease or ≥2-fold increase in abundance, *p* < 0.05). In the study, 81 decreasing and 55 increasing CSPs were observed [41]. The remaining significant differentially abundant proteins were analysed using both SecretomeP and an in-progress version of the LSPpred/SPLpred suite of tools to form a union of candidate UPS proteins. Proteins predicted by either LSPpred or SPLpred were labelled UPS and proteins predicted by only SecretomeP as LSPs were labelled as suggested unconventional secretory proteins (sUPSs). Proteins not predicted by any tool were termed likely not-secretory (Lns). Notably, the low-confidence modes of LSPpred/SPLpred were not included in this work and so we revisit this analysis with the final versions of these tools in order to observe their use in an independent test and consider the consequences of using LSPpred and SPLpred in place of SecretomeP, rather than in addition.

**Figure 4:**
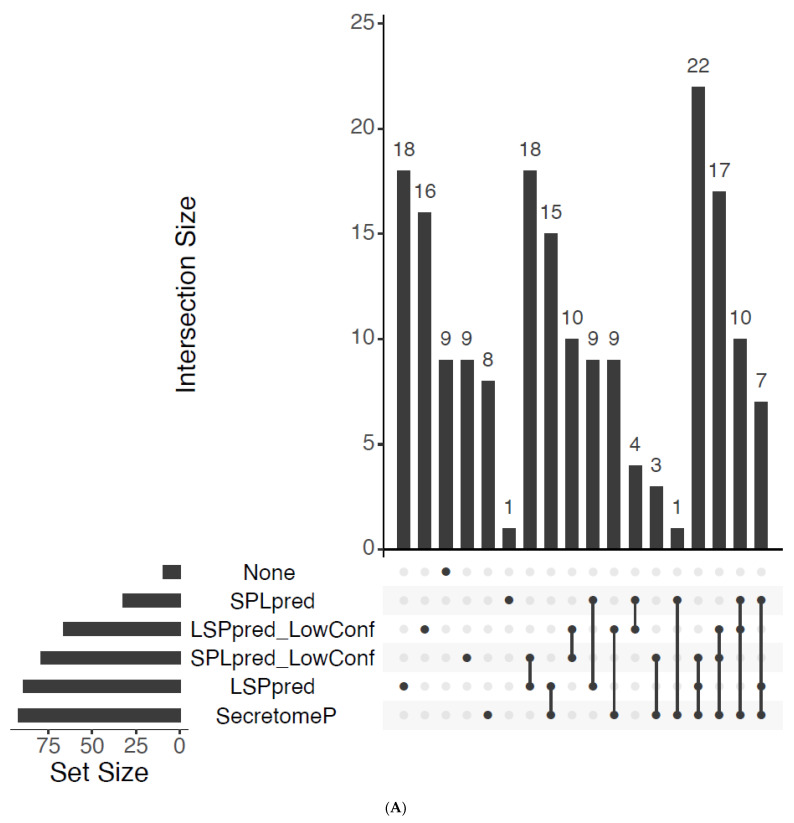

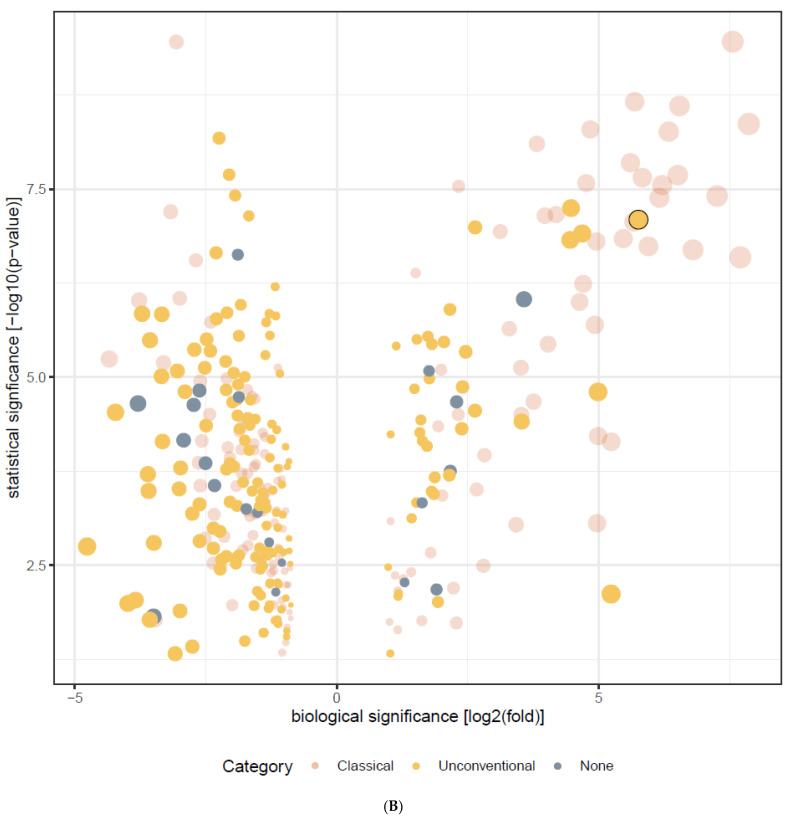
Tomato results. (**A**) UpSet plot of excess manganese secretome results indicating the concordance between predictions of LSPpred and SPLpred (including low-confidence modes), as well as SecretomeP. Agreement between the high- and low-confidence modes is implicit when assigned UPS by the high mode. (**B**) Partial recreation of Figure 2A from Ceballos-Laita [41], with a volcano plot of statistically significant (ANOVA, *p* < 0.05) proteins with changes to abundance are shown. Size is proportional to the log fold change and colour-coded by the new assignment of UPS category through use of all modules of SPLpred and LSPpred (light red = classical secretion, gold = unconventional secretion, grey = no secretion predicted). The Major allergen d 1 (Solyc09g091000.3.1) is marked by a black circle.

Applying the low-confidence modules largely reclassifies sUPS proteins into other categories. Using the low-confidence modes of SPLpred/LSPpred instead of SecretomeP retains 29 of the 37 sUPS proteins previously predicted via only SecretomeP. These 29 are obtained from low-confidence modes of LSPpred (9), SPLpred (3), or both (17) (Figure 4A). The number of proteins without any prediction under the previous strategy is reduced from 44 to 9 due to 35 new low-confidence predictions, 16 from LSPpred, 9 from SPLpred, and 10 from both. Manual adjustments in previous work were made to six predicted sUPS proteins, reclassifying them as Lns [41]. Using the low-confidence cut-off, five are retained as putative UPS proteins. The classification of nine Lns proteins was unchanged. A strategy of replacing SecretomeP with the low-confidence SPLpred and LSPpred modules, therefore, resulted in a net increase from 127 UPS/sUPS to 159 differentially abundant proteins predicted to be UPS proteins. The functional categories of these proteins using the updated classifications are shown in Supplementary Table S2. Proteins no longer considered UPS include one increased abundance protein, Alanine aminotransferase 2, and seven decreasing in abundance consisting of two ribosomal proteins, a photosynthesis protein initially reclassified as Lns, a ferredoxin-thioredoxin reductase, a hop-interacting protein, an ATPase subunit, and a remorin annotated as a protein of unknown function. The additional 35 UPS proteins consist of 9 with increased abundance and 26 with decreased abundance. Notably, four of the nine additional proteins with increased abundance are related to glutathione metabolism, indicating that the expanded LSPpred/SPLpred tools can recognise these proteins related to antioxidant processes. The overlap between proteomes of the xylem and root of tomato under excess Mn was also included, and Major allergen d 1 (Solyc09g091000.3.1) identified as greatly increased in abundance in both studies and a candidate for further work, though it was categorised as Lns in the previous analysis. As one of the more significant proteomic results (Figure 4B, circled), its reclassification as UPS using SPLpred/LSPpred suggests these new tools may be valuable in identifying additional plant LSPs.

Other independent datasets that can be used to explore the effect of LSPpred are experiments involving known LSPs. For example, in a recent publication, a recombinant form of celery MDH was used to capture interacting proteins when its unconventional secretion was induced by salicylic acid treatment [42]. The authors identified 55 Tier 1 proteins that were present in a high number of replicates post-induction. Using both modules, 46 and 28 of 55 Tier 1 proteins were predicted by either SPLpred or LSPpred in low- and high-confidence modes, respectively. For the 78 Tier 2 proteins that appeared in fewer replicates, 59 and 24 were found in low- and high-confidence sets from either tool. Whilst these proteins are MDH-related, it is unclear if all of them are also unconventionally secreted or are part of the mechanism by which MDH is secreted. In either case, application of SPLpred/LSPpred may detect additional UPS-related proteins that can be prioritised in future protein localisation studies for verification purposes and to learn more about the mechanisms of UPS.

## 4. Discussion

### 4.1. Evaluation and Limitations

Evaluating LSP prediction methods, like developing the methods themselves, is challenging without experimentally verified plant LSPs. The prediction tools described in this study are the outcome of research exploring the methodology of protein secretion prediction. Despite the limitation of only a few plant LSPs currently being known, several points can be made by considering the qualities and design of these predictors. Firstly, the LSPDB and both prediction tools did not rely on the limited specific examples of plant LSPs. Instead, the work was built on methods to identify LSPs by their relationship to secretory proteins, either sharing protein features or through protein–protein interactions. Despite the small number of bona fide LSPs, the results in Table 3 reassuringly indicate that these independent methods were able to predict all three known plant LSPs.

In practise, the SPLpred and LSPpred modules are accurate according to the bounds of the training approaches used to build them and each attempt to control for false positives to identify putative LSPs. Each tool also offers distinct hypotheses that emulate the biological context of candidate proteins. SPLpred is based on finding proteins with similarity to known extracellular proteins, under the theory that these similarities imply similar ‘suitability’ to the extracellular environment. In contrast, LSPpred does not directly assume this, allowing LSPs to differ from CSPs, whereby they typically inhabit a different subcellular environment, which may be relevant for moonlighting or stress-related secretion that occurs under specific conditions.

The criteria for selecting the LSPDB outputs are also limited. The confidence criteria for the GO terms and PFAM domains were determined on either exclusivity or majority rather than statistical enrichment in those classes. The possibility that the NONSEC group may contain unobserved LSPs, as well as the uneven PFAM distributions and hierarchy GO terms in those classes, led to these simplified criteria. Evaluating the PPI network by establishing the cut-off for SEC interactions amongst SEC proteins determined the network criteria. The absence of PPI in many proteins, however, may mean that since the threshold is not sufficient to identify all SEC proteins, it is expected to also be limited when applied to the other classes. Applying a similar statistical framework to GO terms, using PPI and PFAM domains to compare ‘baselines’ of CSPs to LSP candidates is an avenue for future iterations of database criteria establishment.

Lack of ‘gold standard’ plant LSP data is the biggest issue for predicting LSPs, as exemplified by this study. Modifying training data to compensate for missing data is a double-edged sword. We have shown its danger when applied without testing for bias. SPLpred tempers bias through training data selection; however, data imbalance is also an issue for machine learning tools. In this case, training data were mostly NONSEC proteins. LSPpred and SPLpred compensate for this imbalance, but there are trade-offs. The lack of positive examples led to the use of aggregated data—data pooled from cell wall proteomes of different tissues, such as cell suspension cultures, roots, apoplastic fluid, etc.—at the cost of tissue specificity.

Despite their limitations, the application of the high- and low-confidence versions of the LSPpred/SPLpred modules to the tomato differential protein abundance dataset [41] does provide insight into the use of the tools in practice. The large degree of concordance between the tools’ predictions and proteins with observed biological change, as well as similar overlap with the results of the still widely used SecretomeP, shows that these plant-trained tools can be used in place of SecretomeP in bioinformatics workflows of plant secretome experiments. The use of computational predictions alone in the discovery of new LSPs will produce many false positives, especially in low-confidence mode. However, coupled with empirical evidence of biological change in response to stimulus or genetic difference, predictions can be a useful guide to prioritise candidate proteins for experimental follow-up.

Notably, though, there is an underlying assumption that LSPs are able to be predicted. Since UPS has several suggested pathways in plants, there may be sequence motifs or other features that are specific to each pathway and, ultimately, a single prediction model may not be appropriate. Pathways may also differ in terms of either active or passive selection of proteins. For example, the uptake of proteins (and small RNAs [17]) into MVBs could contribute to the ‘contaminants’ observed. The approach in this work, however, has been to utilise observed data and to ignore whether the LSP mechanisms are passive or active. With the verification of more candidate LSPs in the future, motifs or mechanisms may be identified, which could allow LSP candidates to be differentiated as passive or active. This would also permit the true positive data associated with a pathway to be collated and used individually for LSP classification rather than in a combined manner that the lack of ’gold standard’ data necessitated in this work.

### 4.2. Insights into Feature Importance

Random forests were chosen as the machine learning model to ensure that some insight into how the models perform was available. Despite the caveats on evaluating the tools, the common and unique features they use to infer their predictions are of interest. The features of importance can be viewed for each prediction tool, and the top 20 features for both LSPpred and SPLpred are shown in Supplementary Figure S4. In total, LSPpred2 and SPLpred3 have 301 and 289 features, respectively, of which 99 are common to both. The Gini importance sums to 1, and the top ranked in each contributes the most to prediction. For each predictor, the features with the highest Gini importance scores are still quite low. “Secondary Structure Transitions 23” contributed 3.3% in SPLpred and “G entropy” 1.5% to LSPpred, respectively. For each predictor, the remaining Gini importance (96.7%, 98.5%) comes from a long tail of other features, suggesting that the models are based on the combination of many protein elements.

Two observations from the highest-ranking features can be made. Firstly, a notable difference between SPLpred and LSPpred is the presence of particular amino acid features in LSPpred, including glycine (G) entropy as the most important and the frequency of G, is also amongst the most important (Supplementary Figure S4). In the ProFET implementation, entropy is calculated in the information theory sense, related to the frequency and probability of observing an amino acid in a sequence. Amino acid features using compressed alphabets are also present in the top 20 features of LSPpred versions, including both asparagine and glycine entropy. Secondly, whilst both predictors contain features of windowed scores based on scaled amino acid properties, SPLpred alone contains in its top features those related to the summarised composition, transitions, and distribution (CTD) syntax [43] of reduced alphabets. The most highly ranked CTD-encoded alphabets represent protein properties, such as polarity, polarizability, charge, solvent accessibility, and secondary structure. The most relevant features for SPLpred are all related to the physical properties of amino acids and relate to secondary structure, which could suggest those structures preferred by secreted proteins are being recognised.

For LSPpred, the inclusion of simple amino acid frequency and entropy-based features of asparagine and glycine residues offers a direction for further work in how the LSP candidates may find their way into the secretory pathway. Both these amino acids are considered targets for the cleavage site of GPI anchors [44]. Another secretory pathway aspect could be *N*-linked glycosylation, which begins in the ER and continues in the Golgi, with glycosylation occurring on asparagine residues in the appropriate context (Asn-X-Ser/Thr, where X is any amino acid except Pro) of the protein. Given that one proposed function of UPS is as a secretory route that avoids PTMs, this and other PTMs related to asparagine and glycine (e.g., acetylation, myristylation (glycine)) could be an area for further investigation with respect to their relationship to LSPs.

### 4.3. Availability and Recommendations for Use

LSPpred is available from http://lsppred.lspdb.org/ (accessed on 7 March 2023). It can either be downloaded and run locally via Docker container for technical users or via a convenient Web interface where it is available for academic use with registration. For the local install, the following data are available. The selected models in this work are written to files using the *joblib* library. Python scripts for LSPpred and SPLpred take FASTA input, convert it to numerical features using ProFET, and then apply the prediction model. For each protein input, the scripts return each individual model probability. Where that probability exceeds the determined threshold, a true prediction is also returned. The consensus between LSPpred and SPLpred is also included. Optionally, low-confidence results between 0.5 and the FPR threshold can also be returned, as well as the consensus result using these values. The tool can be invoked using the Python script lsppred.py. These scripts and the binary *joblib* file to each sequence are available at: https://github.com/LSPtools/LSPpred (accessed on 7 March 2023). The LSPDB database contents are also available at http://lspdb.org/ (accessed on 7 March 2023). A BLAST interface can be accessed at http://blast.lspdb.org/ (accessed on 7 March 2023) to compare input queries against the database categories (i.e., SEC, UNCLASSIFIED, etc.).

These LSPpred tools are based on the model organism *Arabidopsis thaliana*. The applicability to other plant species is expected to be sufficient, especially relative to the mammalian trained SecretomeP and given the promising results present here. As noted in Table 3, LSPpred (and to a lesser extent SPLpred) predicted the plant LSPs of which only one is from *Arabidopsis*. Interestingly, the Gram-positive bacterial tool PeNGaRoo also predicted three of the four LSPs. For species outside the scope of the LSPpred/SPLpred targets, the BLAST interface can be used to give an informative link between the LSPDB training data and queries of interest. There is no intention for either LSPpred or SPLpred to be applied to other organisms outside the scope of the training data. Using *Arabidopsis* proteins as input assumes the properties observed are representative of land plants; however, the possibility of capturing broader conserved mechanisms is worthy of further research. An initial analysis can invert the comparison; that is, rather than observe the Gram+ tool’s predictions on plant LSP data, we apply the plant LSP tools developed here to the training data of other UPS tools (Supplementary Figure S5). Notably, the AUROC and ROC curve for the application of LSPpred to the PeNGaRoo training data is surprisingly high at 0.808. This may be a technical artefact of the two programs, such as the possibility that the training data between the tools are similar, rather than a demonstration of conserved unconventional secretion mechanisms being captured in their models. To determine whether this is the case, CD-Hit-2d (Li and Godzik 2006) was used to establish which proteins in the LSPDB output (high, medium, and low confidence) correspond to the training data. Only 6/141 PeNGaRoo proteins were found with sequence similarity above 0.4 compared to the LSPDB outputs. No similarity at this threshold was found between the LSPDB candidate proteins and the SecretomeP training data. Removing these six proteins from PeNGaRoo improves the result slightly (AUROC to 0.825). Though 63 proteins in the negative set match the LSPDB NONSEC class, and so similarity to negative data could also be an explanation, this result is still unexpected. These similarities in training data offer plausible explanations for the accuracy of LSPpred on bacterial data, though the possibility remains that a conserved mechanism between plant and bacterial LSPs is being recognised. This similarity could be due to the shared property of cell walls amongst these organisms. Further work is required to provide insight into this intriguing observation.

The tools presented here are a less-than-perfect attempt at predicting LSPs in plants. Crucially, the observation of possible LSPs in plant apoplastic proteomic studies is key to the underlying input data. Organism-wide applications of the tool without experimental observation are not recommended. The inherent uncertainty in these tools due to the speculative nature of the input and hypothesis behind their design, as well as the estimated false-positive rates, means all results should be matched with an experimental observation. Genome-wide analysis without experimental evidence is not recommended. Prioritisation of empirical output for further analysis or experimental validation is critical. Functional validation of the mechanism for UPS is the end goal rather than mere observation. With each plant protein found to undergo UPS, the possibility of using verified positive data to train a model increases, moving from ‘shallow’ to ‘deep’ learning. Larger datasets would allow for more powerful deep learning techniques to be employed, such as gradient boosting (e.g., XGBoost), in place of the simple Random Forest tool on small data used here.

Plant LSPs are difficult to identify using either experimental or bioinformatics approaches. The true number of LSPs observed in proteomes has been obscured by the likelihood of intracellular protein contamination, and distinguishing LSPs from contaminants remains a difficulty in analysing experimental results. As prediction tools improve, they can identify more LSPs for validation and start a virtuous cycle that leads to an increase in knowledge regarding the mechanisms and biology behind leaderless protein secretion in plants. With each additional LSP confirmed, this knowledge shall move towards defining protein features leading to secretion, rather than those they lack. At present, the tools presented here can predict likely LSPs and accelerate the discovery of more plant LSPs.

## Figures and Tables

**Table 1:** LSPDB scoring criteria. Individual confidence criteria for each of protein–protein interaction network, PFAM domain, and GO term score for all LSPDB annotations. Overall confidence classification for proteins based on scoring ≥ 5 (High), 3 to 4 (Medium) or less than 3 (Low). An additional criterion of homodimers in the PPI network is classified as 0.5 points to note this category of interest, though this does not affect the scoring. Numbers in brackets correspond to the number of *Arabidopsis* proteins falling into each of the categories.

Confidence (Overall Score)	Network	PFAM Domain	GO Term
Tier 1 (1 point)	All UNCLASSIFIED proteins with SPT interactions matching the profile of SPT with >1 SEC and 0 NONSEC (16 proteins)	PFAMs predominantly in SEC, possibly in SPT, must be in UNCLASSIFIED (15 proteins)	GO predominantly in SEC, possibly in SPT, must be in UNCLASSIFIED (186 proteins)
Tier 2 (2 points)	All UNCLASSIFIED proteins with SEC interactions matching the profile, >1 SEC > 33 NONSEC (89 proteins)	PFAMs exclusive to SEC, SPT and UNCLASSIFIED (1 protein)	GO exclusive to SEC, SPT and UNCLASSIFIED (12 proteins)
Tier 3 (3 points)	All UNCLASSIFIED proteins with SEC interactions matching the profile, >1 SEC ≤ 33 NONSEC (124 proteins)	PFAMs exclusive to SEC and UNCLASSIFIED (5 proteins)	GO exclusive to SEC and UNCLASSIFIED (8 proteins)

**Table 2:** Tool results. Accuracy statistics for each version of LSPpred and SPLpred considered. Preferred models in bold.

Tool	Version	AUROC	Mean Threshold for 0.05 FPR	TPR for Threshold	Balanced Accuracy (Controlled Threshold)	Balance Accuracy (0.5)
LSPpred	1	0.82 ± 0.05	0.65	0.3675	0.6	0.71
**LSPpred**	**2**	**0.84 ± 0.05**	**0.68**	**0.355**	**0.63**	**0.65**
SPLpred	1	0.99 ± 0.01	0.61	0.946	0.95	0.94
SPLpred	2	0.88 ± 0.06	0.70	0.62	0.72	0.81
**SPLpred**	**3**	**0.90 ± 0.05**	**0.70**	**0.62**	**0.76**	**0.82**
SPLpred	4	0.89 ± 0.04	0.67	0.58	0.83	0.84

**Table 3:** LSPDB results. Results of prediction tools applied to the three best plant LSP candidates and bacterial HYG^R^, with the predicted category score shown. Correct predictions of extracellular location for the LSPs are in bold.

Tool	HYG^R^	SAMS2	MDH	Helja
**SecretomeP (Mammalian)**	No 0.513	No 0.421	No 0.450	No 0.447
**SecretomeP (Gram-)**	No 0.078	No 0.173	No 0.108	**Yes** **0.55**
**SecretomeP (Gram+)**	No 0.087	No 0.153	No 0.086	No 0.402
**DeepLoc**	Peroxisome, Soluble 0.386 Extracellular 0.007	Cytoplasm, Soluble 0.730 Extracellular 0.0018	Cytoplasm, Soluble 0.554 Extracellular 0.0042	Cytoplasm, Soluble 0.742 Extracellular 0.0878
**SRTpred**	Non-Secretory Protein −0.362	Non-Secretory Protein −1.5007	Non-Secretory Protein −0.9236	**Secretory Protein** **0.05923**
**ApoplastP**	Non-apoplastic 0.93	Non-apoplastic 0.8	Non-apoplastic 0.82	**Apoplastic** **0.81**
**PeNGaRoo** **(Gram+)**	No 0.417	**Yes** **0.575**	**Yes** **0.626**	**Yes** **0.582**
**OutCyte**	Intracellular 0.9343	Intracellular 0.5631	**UPS** **0.5345**	Intracellular 0.551
**ExoPred**	NA	NA	NA	**Y**
**EffectorP 3.0**	N 0.885	N 0.501	N 0.632	**Y** **0.912**
**SPLpred (high)** **SPLpred (low)**	No No 0.474	**Yes** **Yes** **0.716**	No **Yes** **0.666**	**Yes** **Yes** **0.941**
**LSPpred (high)** **LSPpred (low)**	**Yes** **Yes** **0.714**	**Yes** **Yes** **0.839**	**Yes** **Yes** **0.789**	**Yes** **Yes** **0.690**

## Data Availability

All data is available from the LSPDB database and prediction tool websites: http://lspdb.org/ (accessed on 7 March 2023) and http://lsppred.lspdb.org/ (accessed on 7 March 2023) respectively. Source code and models for the machine learning tools are available from GitHub: https://github.com/LSPtools/LSPpred (accessed on 7 March 2023).

## Supplementary Materials

The following supporting information can be downloaded at: https://www.mdpi.com/article/10.3390/plants12071428/s1, Table S1: Summary of literature included in LSPDB, Table S2: Updated Sol LSP results, Supplementary Methods—LSPDB, Figure S1: SPLpred design, Figure S2: SPLpred comparisons, Figure S3: LSPpred comparisons, Figure S4: Model feature importance, Figure S5: Other tools and data. References [45,46,47,48,49,50,51,52,53,54,55,56,57,58,59,60,61,62,63,64,65,66,67,68,69] are cited in the Supplementary Materials.
