# Supplementary material for: LSPpred Suite: Tools for Leaderless Secretory Protein Prediction in Plants
Source: Plants (Basel). 2023 Mar 23;12(7):1428. doi: 10.3390/plants12071428 (PMC10097205; doi:10.3390/plants12071428)
Supplement: Supplementary file 1 [file plants-12-01428-s001.zip › Supplementary_files_v2.pdf]

<u>Supplementary Tables</u>

Supp Table S1. Summary of literature included in LSPDB

Reference for each paper included in the LSPDB with counts of observed proteins (gene level) indicated.

Basu et al. (2006) [45]	48
Nguyen-Kim et al. (2016) [46]	1968
Herve´et al. (2016) [47]	1662
Chen et al. (2015) [48]	27
Boudart et al. (2005) [49]	101
Haslam et al. (2003) [50]	10
Trentin et al. (2015) [51]	116
Cheng et al. (2009) [23]	75
Charmont et al. (2005) [52]	49
Minic et al. (2007) [53]	104
Kwon et al. (2005) [54]	168
Robertson et al. (1997) [55]	16
Tran and Plaxton (2008) [56]	49
Zhang et al. (2011) [27]	131
Borderies et al. (2003) [57]	153
Irshad et al. (2008) [58]	257
Oh (2005) [59]	78
Bayer et al. (2006) [60]	716
Feiz et al. (2006) [61]	178
Borner (2003) [62]	30
Casasoli et al. (2008) [63]	33

Chivasa et al. (2002) [64]	72
Schultz (2004) [65]	12

Supp Table S2. Updated Sol LSP results (External .xls File)

Protein information for potential UPS proteins after re-evaluation of data from Ceballos-Laita et al [41] using the low confidence modes of LSPpred and SPLpred. Column data taken from Supplementary Tables 3 and 4 of that work, with exception of Reclassification column which indicates whether the protein's UPS status remains positive (Retained), new due to the use of lower thresholds (Low confidence), removed as UPS (Not kept) or unchanged as non-secreted (Unchanged). Proteins predicted as true (predicted to be secreted) then manually adjusted in previous work are noted (adj).

Supplementary Methods – LSPDB

Secretory feature annotation

As a first step to creating a database of a putative LSPs, a bioinformatics workflow was applied to the entire *Arabidopsis thaliana* proteome (Uniprot reference proteome UP000006548) which included collating protein isoform annotation information from prediction programs, observation of a protein's secretion in scientific literature and the experimentally demonstrated interactions between proteins. Protein features relevant to secretion are primarily amino acid motifs with known functions related to CSPs: signal peptides (SP), transmembrane domains (TMDs) and GPI anchor signal sequences. To ensure less well studied proteins have an annotation rather than solely the curated database annotations, a combination of computational predictions for SP (SignalP [36]), TMD (TMHMM [66]) and GPI (BigPi [67] and PredGPI [44]) were applied to the entire proteome. A trade-off for the added level of detail obtained using predictions for all three secretory features is dealing with disagreement between the programs. For example, the similarity between SP and TMD features (with similar hydrophobic properties that prediction tools may confound) can lead to ambiguity, e.g.,

positive predictions for both a SP and TMD in the same N-terminal region. Similarly, overlapping predictions for a GPI anchor and TMD near the C-terminus have been observed. To resolve instances where there is a lack of consensus or where two or more tools predict different features at the same position, a hierarchal approach was used. For each protein, a single positive prediction with either a SP or GPI predictor is sufficient for the protein isoform to be annotated as an SP or GPI accordingly. Where there are multiple predictions between SP or GPI, and TMDs, the range of amino acid positions for each of the SP and GPI predictions is compared to TMD prediction positions. If a TMD prediction overlaps with either SP or GPI predictions, then the TMD prediction is ignored, and the SP or GPI prediction is kept. This weighs SP and GPI features more heavily, but essentially collapses ambiguous predictions into SP-like or GPI-like annotations. This ensures that CSPs can be identified first and reduces the complexity of comparisons between groups.

As the predicted proteome can also include multiple protein isoforms from a single gene, the secretory features may differ between isoforms. We assign an overall secretory status to each protein based on the consistency of the features across each protein isoform. Genes with multiple transcripts with identical secretory features in all isoforms form the core of the LSPDB. Those where at least one of the encoded protein isoforms has a different combination of predicted secretory features compared to other isoforms are labelled as secretory isoforms and labelled separately without further classification. This complexity in secretory features has been observed in other species, including humans [68]). However, as the resolution of proteomic experiments is often limited at the gene level, ambiguity of the secretory features for protein isoforms can limit the ability to determine if secretion is conventional or unconventional or in the case of the LSPDB, which category to assign them to. This ambiguity is not consistent with the requirement to label positive and negative proteins for creating prediction tools, so these secretory isoforms were identified and excluded from the core LSPDB and subsequent prediction tool training data.

Protein classification based on experimental observations

A set of 23 published plant cell wall/secretome proteomic studies (Supplementary Table 1) were analysed for all proteins found according to the criteria for their experimental observation (generally number (and identity) of the peptide fragment(s) specified) and added into the LSPDB. This step explicitly includes proteins dismissed as either contamination due a lack of classical SP or whether they are identified as putative LSPs. Only the observation of the protein is required. In total, 6053 observations of 2677 unique secreted proteins were recorded for *Arabidopsis.* Since the purpose of the LSPDB is to distinguish contaminant proteins from LSPs, observations within the same published paper (i.e., due to multiple experimental conditions) are each counted as observations of that gene.

After annotation of secretory features, the proteins were filtered to exclude proteins with only a TMD, or those with inconsistent features across isoforms. The remaining proteins were then assigned to one of four categories based on the combination of secretory protein features and experimental observations: 1) secretory and observed, 2) secretory and unobserved, 3) non-secretory and observed and 4) non-secretory and unobserved. The first two classification categories are labelled SEC (secretory, 647 proteins) for proteins with a combination of SP, GPI and TMD features that have been observed and NONSEC (non-secretory, 16850 proteins) for proteins that both lack these features and have not been observed in the cell wall secretome papers used in our analysis. The remaining two classification categories are the opposite of these first two classes. The UNCLASSIFIED label (1696 proteins) applies to proteins that have no relevant secretory features yet have been experimentally identified in the LSPDB source studies, noting that the way authors classified these proteins in these studies is not included. These are considered the best pool of candidates for either LSPs or contaminant proteins; further bioinformatics analysis will seek to identify to which of these groups they are most likely to belong. The final classification includes those proteins that possess secretory protein features that have not been experimentally identified as such in the same proteomic studies. We label these as theoretical secretory proteins (SP THEORY or SPT; 2786 proteins).

LSP candidates based on category profiles

To identify LSP candidates within the UNCLASSIFIED category, the following features of protein-protein interaction networks, GO term and PFAM families were used to establish tiered guidelines. Similarity between the secreted and unclassified protein sets was used to identify a set of scoring criteria for PFAM, GO term and PPI features from AtPIN [69] that are either exclusively or predominantly seen in secretory proteins (Table 1). Criteria for PFAM and GO terms were based on those either exclusive to or predominantly seen in SEC and SPT categories (Figure 2). Unclassified proteins that contain these features were then selected. Plotting the network interactions (Figure 2C) suggests, in general, that secretory proteins interact with greater numbers of other secretory proteins, and fewer non-secretory proteins, relative to non-secretory proteins. Using this observation, an absolute cut-off of 33 proteins was used to establish the number of interactions a potential LSP can have with non-secretory proteins. For each line of evidence, a 3-2-1 point-scoring method was used, and each protein scored across each criterion. Overall confidence classification for proteins was based on scoring >=5 (High confidence), 3 to 5 (Medium confidence) or 1 to 3 (Low confidence) from the point method. These cut-offs ensured that higher confidence LSP candidates met multiple feature criteria.

Supplementary Figures

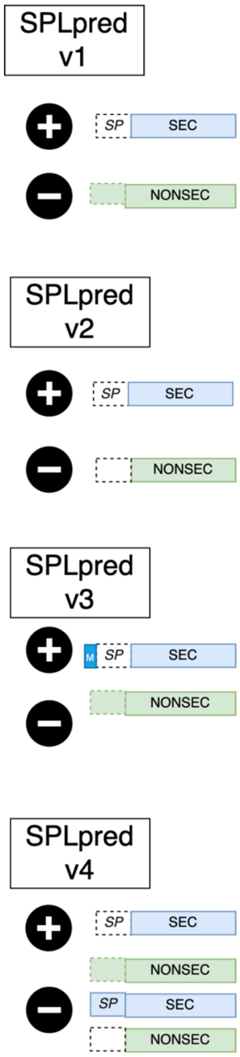

Supp Figure S1. SPLpred design

Overview of four SPLpred versions according to inputs from the LSPDB. Positive and negative data for each version is denoted by the + or – symbol next to the set. Sequence level modifications are indicated by dotted lines (removal of SP region or equivalent length) and boxed M (addition of methionine after removal of SP region).

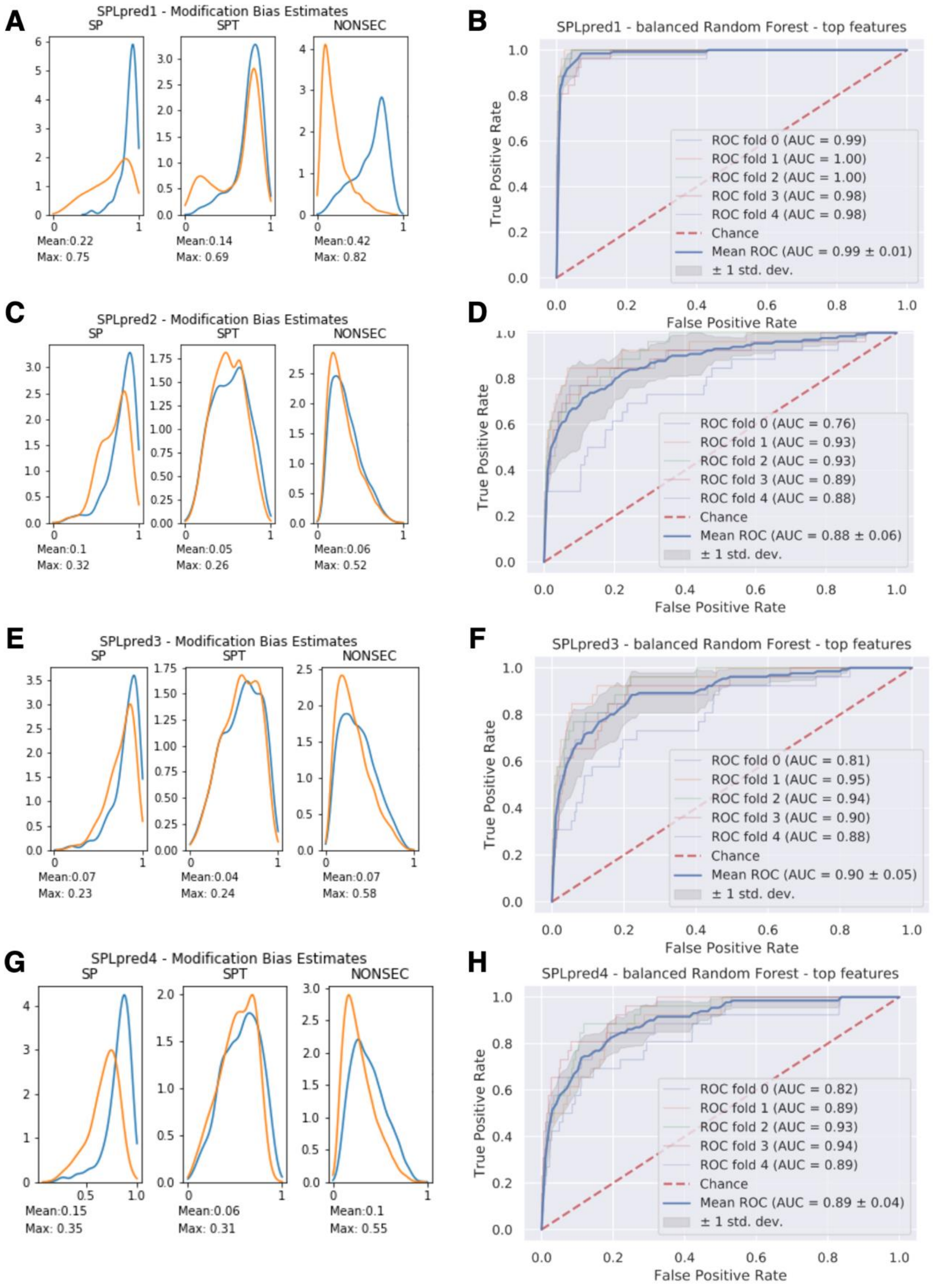

Supp Figure S2. SPLpred comparisons

(A,C,E,G) Modification bias estimates illustrating the density estimates and mean and maximum differences between modified (blue) and unmodified (orange) data (middle) for each SPLpred version 1 through 4. (B,D,F,H) Cross validated ROC scores and mean ROC (for alternative versions. Superior

performance of SPLpred v1 comes with a high bias due to the modifications, whereas SPLpred v3 with modified positive input data and unchanged negative data does not exhibit a shift and has lowest overall bias.

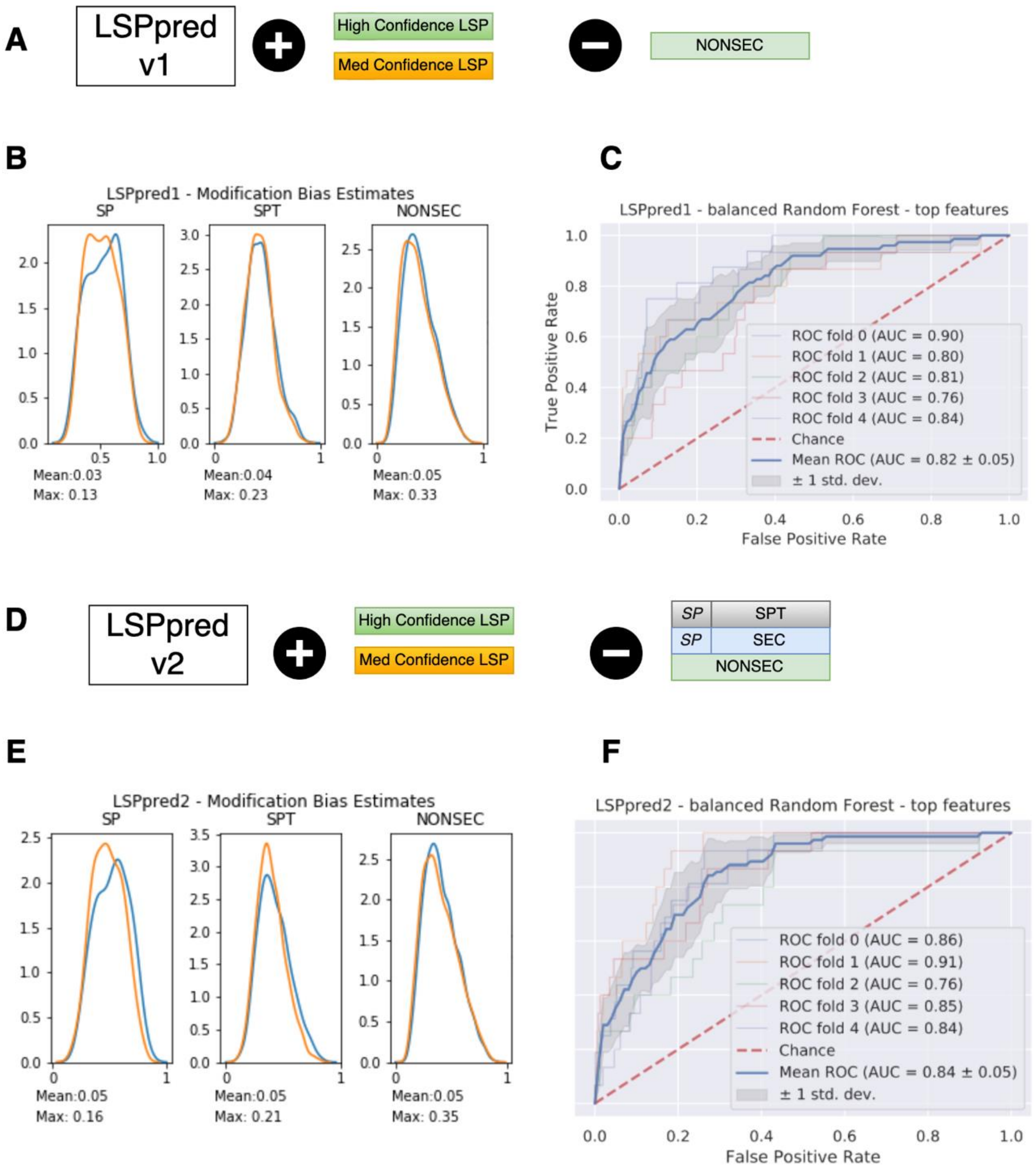

Supp Figure S3. LSPpred comparisons

(A, D) Overview of two versions of LSPpred (v1 and v2) according to inputs from the LSPDB. (B,E) Modification bias estimates for v1 and v2 demonstrating a baseline estimate of the differences

between modified to mimic the removal of SP regions (blue) and unmodified (orange) data (left). (C,F) Cross-validated ROC scores and mean ROC for v1 and v2. Both models perform similarly but LSPpred v2 is selected as SP-containing proteins form part of the negative data set.

Supp Figure S4. Model feature importance

A)

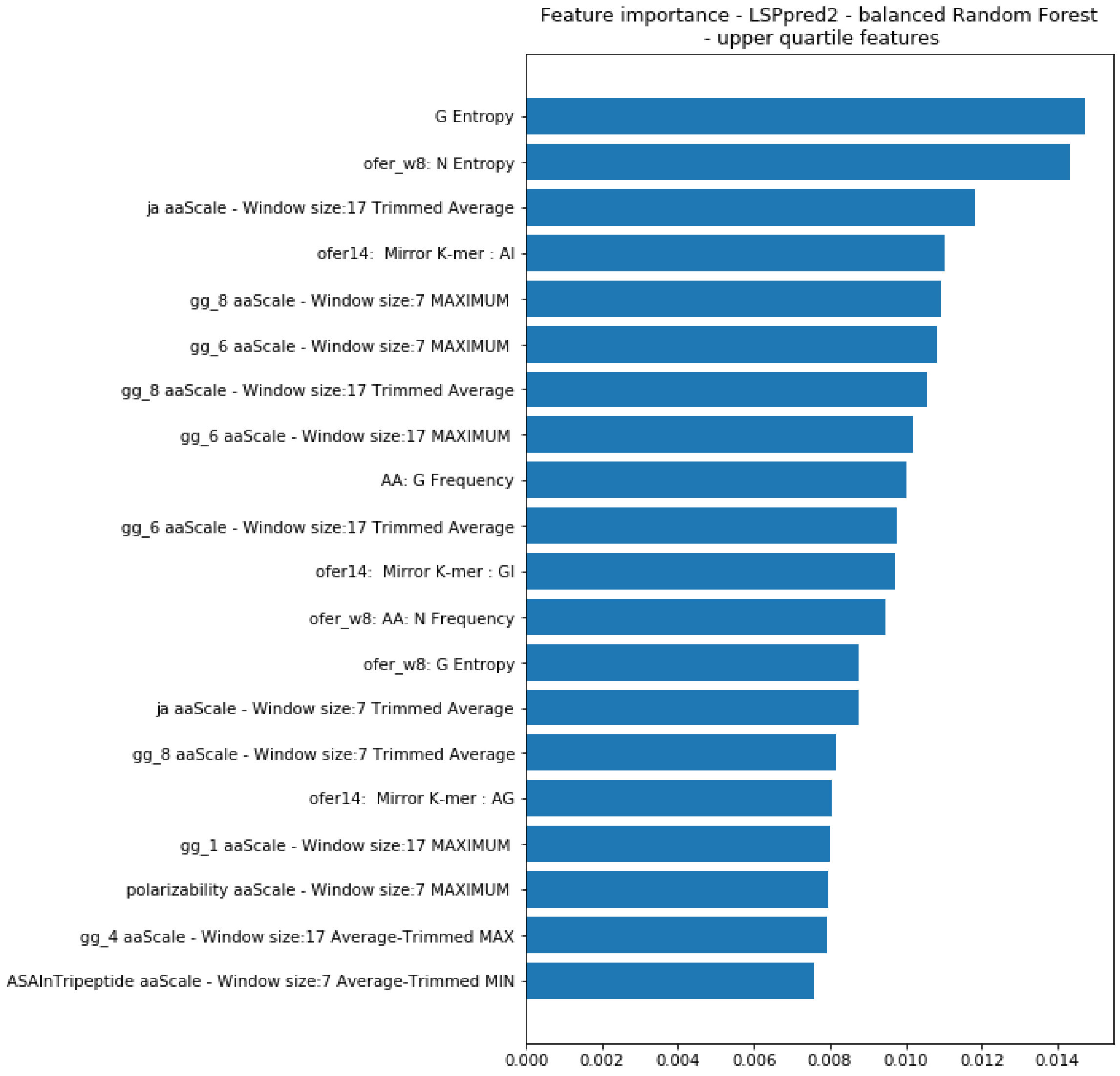

B)

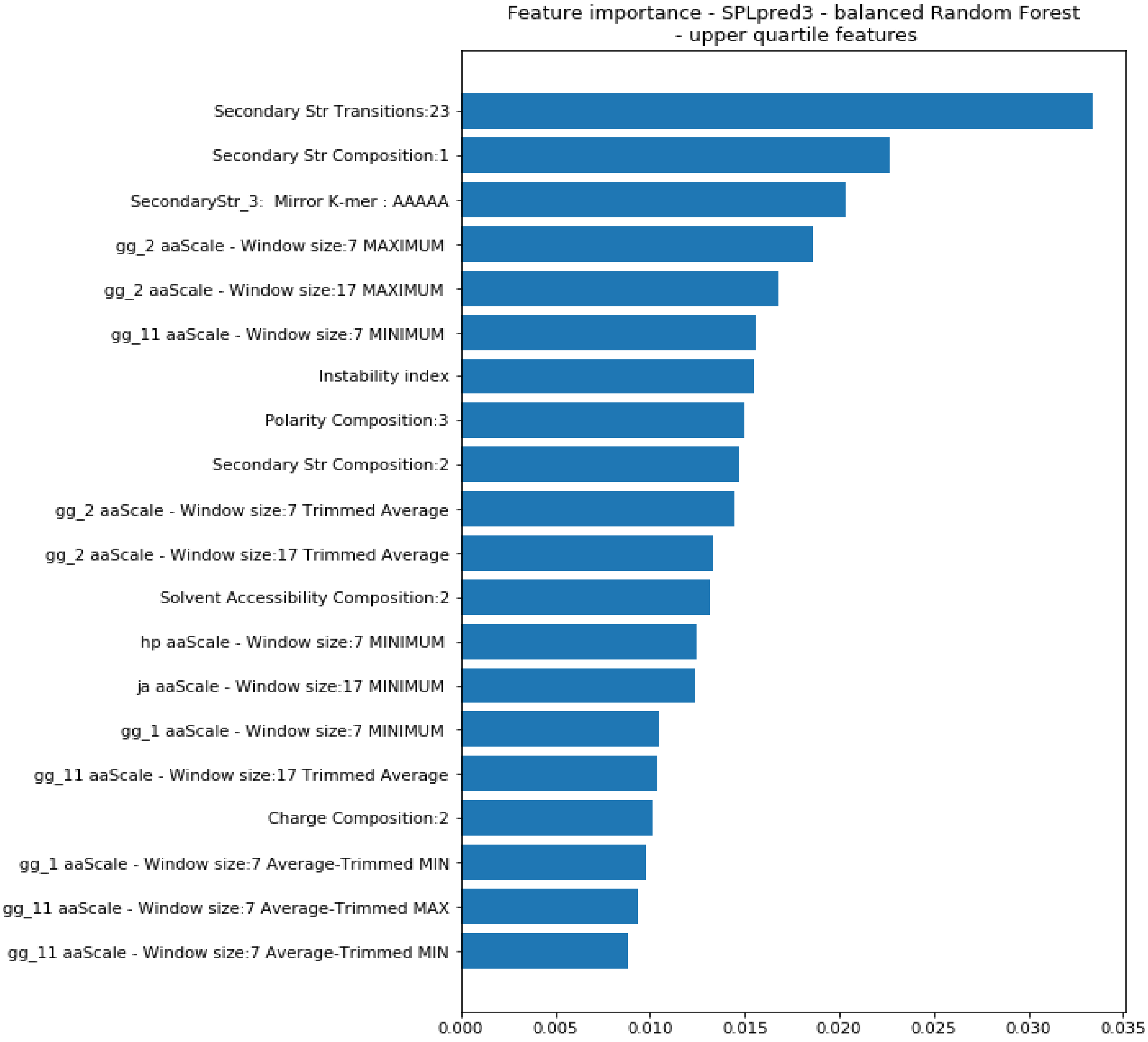

Ranked feature importance by Gini importance score for (A) LSPpred2 and (B) SPLpred 3 random forest models, with features labelled according to the ProFet categories.

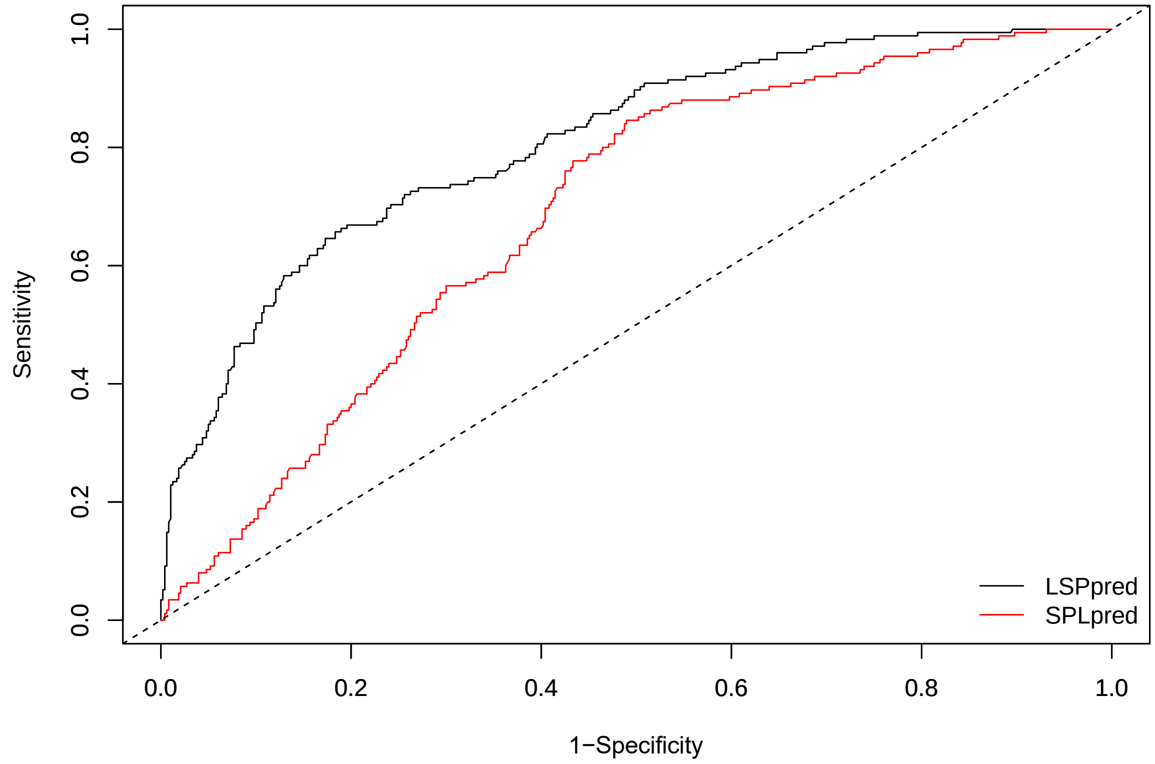

Supp Figure S5. Other tools and data

ROC curves for LSPpred (AUROC 0.808) and SPLpred (AUROC 0.688) applied to bacterial training data from PeNGaRoo [15].
